# A prediction model of risk factors of poor wound healing after craniocerebral surgery

**DOI:** 10.12669/pjms.39.6.7963

**Published:** 2023

**Authors:** Chunlian Zhong, Wei Lu, Wenzhong Xie, Wei Jiao

**Affiliations:** 1 Chunlian Zhong, Department of Neurosurgery, The 904th Hospital of the Joint Logistics Support Force of the PLA, Wuxi 214000, Jiangsu Province, P.R. China; 2 Wei Lu, Department of Neurosurgery, The 904th Hospital of the Joint Logistics Support Force of the PLA, Wuxi 214000, Jiangsu Province, P.R. China; 3 Wenzhong Xie, Department of Neurosurgery, The 904th Hospital of the Joint Logistics Support Force of the PLA, Wuxi 214000, Jiangsu Province, P.R. China; 4 Wei Jiao, Department of Neurosurgery, The 904th Hospital of the Joint Logistics Support Force of the PLA, Wuxi 214000, Jiangsu Province, P.R. China

**Keywords:** Craniocerebral surgery, Prediction model, Risk factors, Wound healing

## Abstract

**Objective:**

To explore the independent risk factors of poor wound healing after craniocerebral surgery, and to generate a risk prediction model.

**Methods:**

A single-center retrospective observational analysis of 160 patients who underwent craniocerebral surgery in The 904th Hospital of the Joint Logistics Support Force of the PLA from February 2018 to February 2021 was carried out. Patients were divided into Group-A (n=70) and Group-B (n=90) according to postoperative wound healing outcome. Logistic regression was used to analyze the independent risk factors, and a nomogram prediction model was constructed using R software. The receiver operating characteristic (ROC) curve was used to test the predictive ability of the model, and the fitting effect was verified by Hosmer Lemeshow.

**Results:**

The duration of operation, surgical site infection, diabetes mellitus, and the time of intubation in Group-B were significantly lower than Group-A (*P*<0.05). Serum albumin (ALB) and hemoglobin (HGB) in Group-B were significantly higher than those in Group-A (*P*<0.05). Logistic regression analysis showed that long operation duration, surgical site infection, duration of drainage tube, ALB <35g/L, and abnormal HGB were independent risk factors for poor wound healing (*P*<0.05). The area under the ROC curve (AUC) predicted by the model was 0.932, 95%CI (0.862~1.000). The Hosmer-Lemeshow goodness of fit test showed that the expected probability calculated by the model matched the actual probability (*P*>0.05).

**Conclusions:**

Long operation duration, surgical site infection, duration of drainage tube, ALB <35g/L, and abnormal HGB were risk factors for poor wound healing. The nomograph model based on these factors showed good discrimination, calibration, and clinical effectiveness in predicting poor wound healing.

## INTRODUCTION

Impaired healing of surgical incisions is the main factor which impacts the speed of patient recovery after an operation.^1^ In craniocerebral surgery, postoperative wound nonunion is common, and manifests as infection or necrosis of the wound, with possible cerebrospinal fluid leakage.^2^,^3^ Poor wound healing after craniocerebral surgery not only increases hospitalization time and the economic burden, but also seriously affects quality of life of patients, which has become a problem that neurosurgeons and nurses often face.^4^,^5^

There are various factors which increase the likelihood of poor wound healing after craniocerebral surgery, such as old age, diabetes mellitus, long operation time, inappropriate utilization of antibiotics, and postoperative hypoproteinemia. These factors are complex, modifiable and are easily ignored.^6^sequential physiologic process that results in timely healing with full re-epithelialization, resolution of drainage, and return of function to the affected tissue. Chronic wounds, however, do not follow this sequence of events and can challenge the most experienced clinician if the underlying factors that are impairing wound healing are not identified. The purpose of this article is to present recent information about factors that impair wound healing with the underlying pathophysiological mechanism that interferes with the response to tissue injury. These factors include co-morbidities (diabetes, obesity, protein energy malnutrition Therefore, the establishment of a clinical prediction model with high diagnostic efficiency is important for early diagnosis of impaired wound healing or the timely prediction of the prognosis.^7^ The prediction model may help to guide the generation of symptomatic treatment and rehabilitation nursing programs and prevent the malignancy of the disease and possible complications.^8^ However, at present, there is a scarcity of existing English scientific literature to build a prediction model for poor wound healing after craniocerebral surgery.

While there are studies on risk factors of wound healing, very few of them focus on wound healing after craniocerebral surgery. The main goal of this study is to explore the independent risk factors for the occurrence of poor wound healing after craniocerebral surgery and to establish a risk prediction model. Prediction model with high diagnostic prediction efficacy may provide targeted prevention and treatment methods for reducing the adverse risk of wound healing in clinical practice.

## METHODS

Clinical records of 160 patients (91 males and 69 females), undergoing craniocerebral surgery in The 904th Hospital of the Joint Logistics Support Force of the PLA from February 2018 to February 2021, were retrospectively collected for this single-center retrospective observational study. A mean age of the patients was 61.94 ± 7.85 years. Based on the record of wound healing after the operation, 70 patients with poor wound healing were considered as Group-A, 90 patients had good wound healing after operation were considered as Group-B.

### Inclusion criteria:

- Patients undergoing craniocerebral surgery.
- Patients with complete wound healing follow-up records.
- Age ≥ 18 years.

### Exclusion criteria:

- Patients with heart, brain, kidney, and other serious diseases.
- Complicated with coagulation dysfunction and immune system diseases.
- Patients with malignant tumors.

### Ethical Approval:

This study was approved by the Medical Ethics Committee of our hospital (Approval No. 20221213; Date:2022-12-19).

### Clinical indicators:

Information on age, gender, duration of operation, surgical site infection, smoking history, hypertension, diabetes mellitus, duration of drainage tube, serum albumin (ALB), and hemoglobin (HGB) were recorded. Surgical site infection was characterized by redness and swelling of the skin around the incision, pain with purulent secretions, especially multiple infections and multi drug resistant bacterial infections, which can be obtained by querying the medical history.^9^

### Wound healing after discharge:

Medical history follow-up record were collected or obtained through WeChat or telephone follow-up; photos were taken for image recording. Laboratory index measurement: after the operation, patients were fasted and 3ml of venous blood was drawn. An automatic biochemical analyzer (Hitachi 7180, manufacturer: Hitachi Diagnostic Products (Shanghai) Co., Ltd.) was used to detect the content of ALB by immunoturbidimetry. HGB content was determined by SDS Hb method. ALB normal value ≥ 35 g/L.^10^ The normal range of HBG is 131~172g/L. 11

### Statistical analysis:

SPSS 26.0 statistical software was used to analyze the data. The counting data were expressed by frequency, percentage (%) and constituent ratio (%), and the comparison between groups was performed by χ^2^ test. The measurement data were expressed by mean ± standard deviation (*χ̅*±*S*), and the comparison between groups was performed by t test. Logistic regression analysis was used to analyze the risk factors of poor wound healing. The ROC curve was used to test the predictive ability of the risk prediction model for poor wound healing. The Hosmer Lemeshow test verified the fitting effect. *P*<0.05 in all statistics indicates that the difference is statistically significant.

## RESULTS

There were 70 patients (39 males and 31 females) in the Group-A, with an average age of 62.08 ± 7.52 years, and 90 patients (52 males and 38 females) in the Group-B, with an average age of 61.83 ± 8.14 years.

As summarized in Table-I, there was no significant difference in age, sex or history of hypertension between the two groups (*P*>0.05). The duration of operation, incidences of surgical site infection, diabetes mellitus, and the duration of drainage tube use in Group-B were significantly lower than Group-A (*P*<0.05). The ALB and HGB in Group-B were significantly higher than those in Group-A (*P*<0.05; Table-I).

**Table-I:** Single factor analysis of poor wound healing after craniocerebral surgery.

Factor	Group-A (n=70)		Group-B (n=90)		χ^2^	P
Age (>65 years)	21	30%	28	31.11%	0.023	0.880
Sex (Male)	39	55.71%	52	57.78%	0.068	0.794
Duration of operation (>3 hours)	33	47.14%	13	14.44%	27.848	<0.001
Presence of incision infection	36	51.43%	17	18.89%	18.820	<0.001
Positive history of smoking history	20	28.57%	25	27.78%	0.012	0.912
Positive history of hypertension	23	32.86%	28	31.11%	0.055	0.814
Positive history of diabetes mellitus	34	48.57%	23	25.56%	9.095	0.030
Duration of drainage tube (>24 hours)	39	55.71%	16	17.78%	34.569	<0.001
ALB <35 g/L	44	62.86%	16	17.78%	46.296	<0.001
Abnormal HGB	37	52.86%	14	15.56%	34.111	<0.001

ALB: Serum albumin; HGB: Hemoglobin.

The duration of operation, surgical site infection, diabetes mellitus, duration of drainage tube use, ALB and HGB were subjected to the independent variable assignment and multicollinearity test of logistic regression analysis. The tolerance of each factor was >0.1, and the variance expansion factor (VIF) was <10, indicating that there was no multicollinearity between each factor (Table-II).

**Table-II:** Assignment of independent variables and multicollinearity test of logistic regression analysis.

Independent variable	Assignment mode	Tolerance	Variance expansion factor
Duration of operation (hour)	≤3=0, >3=1	0.975	1.026
Presence of incision infection	No=0, Yes=1	0.903	1.108
Positive history of diabetes mellitus	No=0, Yes=1	0.892	1.121
Duration of drainage tube >24 hours	No=0, Yes=1	0.945	1.058
ALB	Normal=0, Abnormal=1	0.930	1.075
HGB	Normal=0, Abnormal=1	0.894	1.119

ALB: Serum albumin; HGB: Hemoglobin.

**Fig.1:**
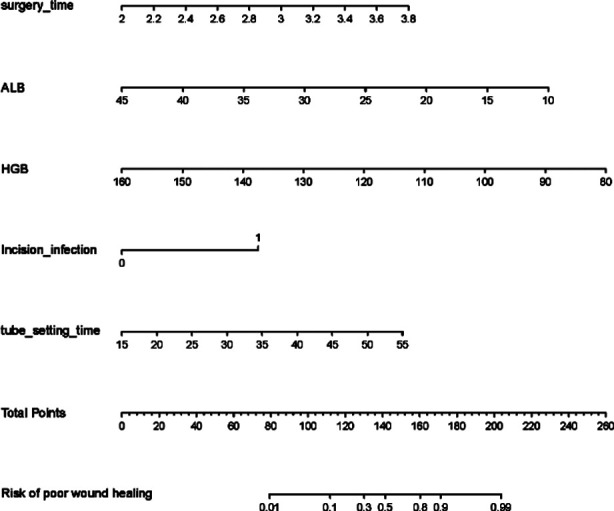
Nomogram prediction model for poor wound healing.

The results of the multivariate logistic regression model analysis showed that long duration of operation, surgical site infection, long duration of drainage tube use, abnormal ALB and HGB were independent risk factors for poor wound healing after craniocerebral surgery (Table-III). The nomogram prediction model was constructed based on the operation duration, surgical site infection, tube insertion time, ALB and HGB, the C index was 0.938 (0.894~0.982; Fig.1).

**Table-III:** Multifactor logistic regression analysis of poor wound healing after craniocerebral surgery.

Variable	Regression coefficient	Standard error	Wald χ^2^	P	EXP(B)	95%CI
Duration of operation	2.456	0.574	18.307	<0.001	11.658	3.785~35.911
Presence of incision infection	1.329	0.571	5.413	0.020	3.776	1.233~11.586
Duration of drainage tube	1.707	0.569	8.985	0.003	5.510	1.805~16.819
ALB	2.563	0.604	18.021	<0.001	12.974	3.973~42.361
HGB	2.955	0.610	23.491	<0.001	19.196	5.812~63.404

ALB: Serum albumin; HGB: Hemoglobin.

**Fig.2:**
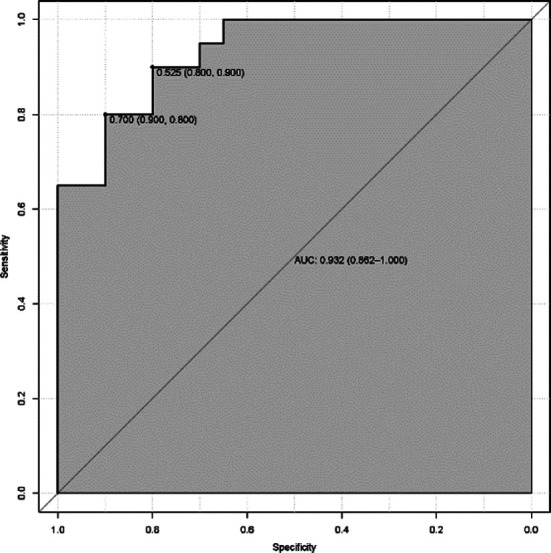
ROC curve of the nomogram model predicting incision healing after craniocerebral surgery.

**Fig.3:**
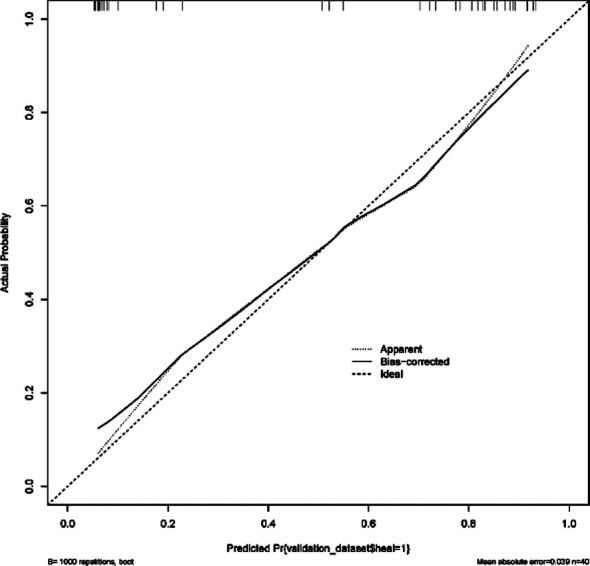
Predicted risk value of poor wound healing after craniocerebral surgery.

The area under the ROC curve predicted by the nomogram model for wound healing after craniocerebral surgery was 0.932, 95% CI (0.862~1.000; Fig.2). The correction curve of the nomogram model for predicting incision healing after craniocerebral surgery tended to be an ideal curve (Fig.3). The results of Hosmer Lemeshow goodness of fit test showed that the nomogram model had good consistency in predicting the risk of poor wound healing after craniocerebral surgery (*χ^2^* =10.996, *P*=0.202).

## DISCUSSION

The results of this study showed that the incidence of poor wound healing after craniocerebral surgery was 43.75% (70/160). Long operation duration, surgical site infection, long duration of drainage tube use, abnormal ALB and HGB were all risk factors for poor wound healing. We constructed a nomogram prediction model and showed that it had good consistency in predicting the risk of poor wound healing after craniocerebral surgery.

Craniocerebral trauma is a relatively large operation. If the operation time is too long, it will cause damage to the tissues near the wound, which will lead to a decline in the tolerance to bacteria, leading to an increase in the probability of postoperative wound infection.^12^ The comprehensive review by Thapa *et al*^13^*oxidation, photolysis* have shown that wound infection is the main factor affecting wound healing. Our results are consistent with these findings.

Factors, such as subcutaneous effusion, wound dehiscence, or postoperative cerebrospinal fluid leakage may lead to wound infection. Therefore, necrotic tissue should be removed in a timely manner, combined with early administtration of antibiotics to reduce wound infection. Additionally, close attention should be paid to postoperative wound healing. Faust *et al*^14^ showed that retention of the drainage tube after the emergency operation of a brain injury is associated with a high incidence of wound infection and poor wound healing. In agreement with this observation, our results also demonstrated that longer drainage tube use was a risk factor of poor wound healing in our cohort of patients. Therefore, in cases of patients with drainage tubes, special measures, such as improved posture and combined antibiotics should be taken to prevent postoperative surgical site infection.

Our results also indicated that abnormal ALB and HGB were risk factors for poor wound healing after craniocerebral surgery. In general, ALB <35 g/L is considered abnormal. Hypoalbuminemia can lead to the reduction of gel osmotic pressure, and is more likely to result in tissue fluid leakage, causing local edema or rupture of the incisions. Additionally, the increase of exudates at the surgical incision makes it possible for pathogens around the incision and on the surface of the skin to invade, thus increasing the risk of poor wound healing.^15^ Low HGB indicates that the oxygen carrying function of the blood is reduced, which may lead to ischemia and hypoxia of the incision tissue, delaying wound healing.^16^and tissue hypoxia is a common issue in such wounds. Granulox (SastoMed GmbH, Georgsmarienhütte, Germany Therefore, nutritional support should be strengthened after operation to prevent hypoalbuminemia and malnutrition.^17^

A national database study by Bitzer *et al*^18^ found that diabetes mellitus is also an important risk factor for retarding postoperative wound healing. The results of our study showed that diabetes mellitus was not a risk factor for poor wound healing in craniocerebral surgery. This discrepancy between our results and the previous data may be due to the fact that the number of patients with diabetes mellitus in the Group-B (good wound healing) was significantly lower than that of Group-A (poor wound healing). It is plausible that the smaller sample size and a single-center nature of the study may have led to a certain bias in the results.

Based on the independent risk factors of poor wound healing after craniocerebral surgery, identified in this study, a nomogram risk prediction model was constructed and visualized in the form of scores. Our results showed that the area under the ROC curve, predicted by the nomogram model for poor wound healing after craniocerebral surgery, was 0.932, 95% CI (0.862~1.000). The correction curve of the nomogram model for predicting incision healing after craniocerebral surgery tends to be an ideal curve. The results of the Hosmer Lemeshow goodness of fit test showed that the nomogram model had good consistency in predicting the risk of poor wound healing after craniocerebral surgery. After some validation, our nomogram model was found to be efficient and accurate, and may, therefore, provide reference for the clinical diagnosis and treatment of poor wound healing after craniocerebral surgery.

### Limitation of the study:

It was a retrospective single center study. Our prediction model was not validated externally with the standard population in other centers, which may lead to some bias in the conclusions. Therefore, the practical value of the nomogram model still needs to be verified by additional multi-center studies with larger sample size.

## CONCLUSION

The results of our univariate and multivariate logistic regression analysis showed that long operation duration, surgical site infection, long duration of drainage tube, abnormal ALB and HGB were all independent risk factors of poor wound healing after craniocerebral surgery. The nomograph model that was constructed based on these risk factors has good accuracy, differentiation and consistency in predicting wound healing after craniocerebral surgery, and may be used in clinical practice.

### Authors’ contributions:

**CZ:** Conceived and designed the study.

**WL**, **WX** and **WJ:** Collected the data and performed the analysis.

**CZ:** Was involved in the writing of the manuscript and is responsible for the integrity of the study.

All authors have read and approved the final manuscript.
